# *N*-Saccharinylmethyl ether

**DOI:** 10.1107/S1600536810010317

**Published:** 2010-03-27

**Authors:** Waseeq Ahmad Siddiqui, Yasmeen Akhtar, Muhammad Akmal, Hamid Latif Siddiqui, Masood Parvez

**Affiliations:** a Department of Chemistry, University of Sargodha, Sargodha, Pakistan; b Institute of Chemistry, University of the Punjab, Lahore 54590, Pakistan; c Department of Chemistry, The University of Calgary, 2500 University Drive NW, Calgary, Alberta, Canada T2N 1N4

## Abstract

In the title mol­ecule [systematic name: 1,1,1′,1′-tetra­oxo-2,2′-(oxydimethyl­ene)bi(1,2-benzothia­zol-3-one)], C_16_H_12_N_2_O_7_S_2_, the benzisothia­zole ring systems are individually planar [maximum deviations of 0.0497 (13) and 0.0195 (19) Å] and their mean planes are inclined at a dihedral angle of 62.76 (4)°. The crystal structure is stabilized by weak inter­molecular C—H⋯O inter­actions. Two O atoms bonded to two S atoms and four aryl H atoms belonging to two symmetry-related mol­ecules lying about an inversion center form a hydrogen-bonded 10-membered ring with graph-set notation *R*_4_^2^(10).

## Related literature

For the biological activity of saccharin derivatives, see: Plath *et al.* (1998 ▶); Salzburg *et al.* (1987 ▶); Kapui *et al.* (2003 ▶). For the synthesis of saccharin derivatives, see: Ahmad *et al.* (2010 ▶); Siddiqui *et al.* (2010 ▶). For related structures, see: Ahmad *et al.* (2009 ▶); Gul *et al.* (2010 ▶); Khalid *et al.* (2010 ▶); Siddiqui *et al.* (2007 ▶, 2008 ▶). For the graph-set notation of hydrogen-bonding patterns, see: Bernstein *et al.* (1995 ▶).
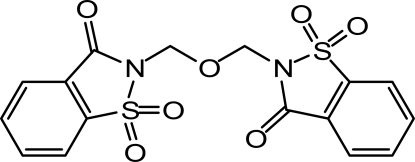


## Experimental

### 

#### Crystal data

C_16_H_12_N_2_O_7_S_2_
*M* *_r_* = 408.40
Monoclinic, 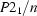

*a* = 8.9317 (4) Å
*b* = 18.3681 (6) Å
*c* = 10.1942 (5) Å
β = 93.517 (2)°
*V* = 1669.29 (12) Å^3^
*Z* = 4
Mo *K*α radiation
μ = 0.37 mm^−1^
*T* = 200 K
0.08 × 0.06 × 0.04 mm

#### Data collection

Nonius KappaCCD diffractometer
Absorption correction: multi-scan (*SORTAV*; Blessing, 1997 ▶) *T*_min_ = 0.971, *T*_max_ = 0.986
6489 measured reflections
3756 independent reflections
3052 reflections with *I* > 2σ(*I*)
*R*_int_ = 0.033

#### Refinement

*R*[*F* ^2^ > 2σ(*F* ^2^)] = 0.049
*wR*(*F* ^2^) = 0.112
*S* = 1.09
3756 reflections
244 parameters
H-atom parameters constrained
Δρ_max_ = 0.30 e Å^−3^
Δρ_min_ = −0.42 e Å^−3^

### 

Data collection: *COLLECT* (Hooft, 1998 ▶); cell refinement: *DENZO* (Otwinowski & Minor, 1997 ▶); data reduction: *SCALEPACK* (Otwinowski & Minor, 1997 ▶); program(s) used to solve structure: *SHELXS97* (Sheldrick, 2008 ▶); program(s) used to refine structure: *SHELXL97* (Sheldrick, 2008 ▶); molecular graphics: *ORTEP-3 for Windows* (Farrugia, 1997 ▶); software used to prepare material for publication: *SHELXL97*.

## Supplementary Material

Crystal structure: contains datablocks global, I. DOI: 10.1107/S1600536810010317/lh5013sup1.cif

Structure factors: contains datablocks I. DOI: 10.1107/S1600536810010317/lh5013Isup2.hkl

Additional supplementary materials:  crystallographic information; 3D view; checkCIF report

## Figures and Tables

**Table 1:** Hydrogen-bond geometry (Å, °)

*D*—H⋯*A*	*D*—H	H⋯*A*	*D*⋯*A*	*D*—H⋯*A*
C1—H1⋯O4^i^	0.95	2.39	3.307 (4)	162
C2—H2⋯O4^ii^	0.95	2.53	3.293 (3)	138
C8—H8*B*⋯O4^iii^	0.99	2.52	3.027 (3)	111

Symmetry codes: (i) 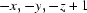; (ii) 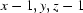; (iii) 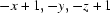.

**Fig. 1.**
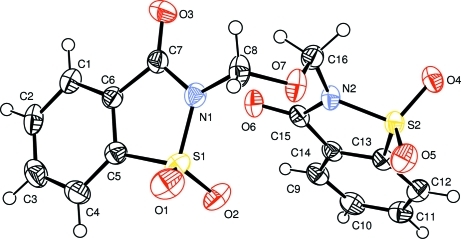
The title molecule with the displacement ellipsoids plotted at 50% probability level (Farrugia, 1997).

**Fig. 2.**
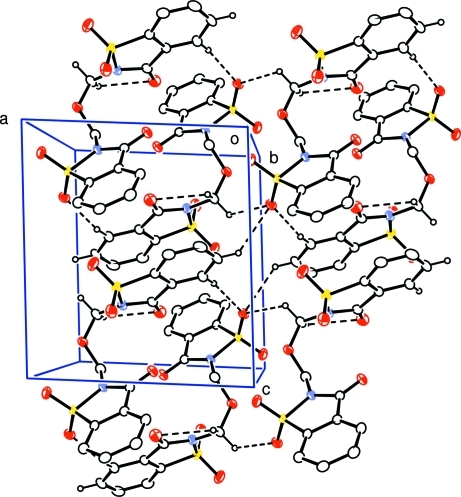
Unit cell packing of the title compound showing hydrogen bonds by dashed lines; the H-atoms not involved in H-bonds have been excluded for clarity.

## supplementary crystallographic information

### Comment

The derivatives of saccharin have found applications as bioactive substances (Plath *et al.*, 1998; Salzburg *et al.*, 1987). They are considered to be the most potent orally active human leucocyte elastase (HLE) inhibitors for the treatment of asthma and other inflammatory diseases (Kapui *et al.*, 2003). Continuing our investigations in the synthesis and development of new saccharin derivatives (Ahmad *et al.*, 2010; Siddiqui *et al.*, 2010) with medicinal potentials, we now report the crystal structure of a novel compound in this paper.

The title compound is presented in Fig. 1. The benzisothiazole ring systems are individually planar with maximum deviations being 0.0497 (13) and 0.0195 (19) Å for S1 and C15 atoms from the mean-planes S1/N1/C1—C7 and S2/N2/C9—C15, respectively; the mean-planes are inclined at 62.76 (4)° with respect to each other. The structure is devoid of classical hydrogen bonds. However, intramolecular and intermolecular interactions of the type C—H···O are present in the structure. Two oxygen atoms bonded to two S atoms and four aryl hydrogen atoms belonging to two symmetry related molecules lying about inversion center form a hydrogen bonded ten membered ring which may be described in the graph set notation as R_4_^2^(10) (Bernstein *et al.*, 1995); details have been given in Tab. 1 and Fig. 2.

The bond distances and angles in the title molecule agree well with the cortresponding bond distances and angles reported in closely related compounds (Ahmad *et al.*, 2009; Gul *et al.*, 2010; Khalid *et al.*, 2010; Siddiqui *et al.*, 2007; 2008).

### Experimental

The synthesis of the title compound will be reported in a future paper. Suitable crystals of the title compound were grown from a solution of CHCl_3_ by slow evaporation at room temperature.

### Refinement

Though all the H atoms could be distinguished in the difference Fourier map the H-atoms were included at geometrically idealized positions and refined in riding-model approximation with the following constraints: C—H distances were set to 0.95 and 0.99 Å, for aryl and methylene H-atoms, respectively, and *U*_iso_(H) were allowed at 1.2*U*_eq_(C). The final difference map was essentially featurless.

### Crystal data

**Table:**

C_16_H_12_N_2_O_7_S_2_	*F*(000) = 840
*M**_r_* = 408.40	*D*_x_ = 1.625 Mg m^−^^3^
Monoclinic, *P*2_1_/*n*	Mo *K*α radiation, λ = 0.71073 Å
Hall symbol: -P 2yn	Cell parameters from 3299 reflections
*a* = 8.9317 (4) Å	θ = 1.0–27.4°
*b* = 18.3681 (6) Å	µ = 0.37 mm^−^^1^
*c* = 10.1942 (5) Å	*T* = 200 K
β = 93.517 (2)°	Block, colorless
*V* = 1669.29 (12) Å^3^	0.08 × 0.06 × 0.04 mm
*Z* = 4	

### Data collection

**Table:**

Nonius KappaCCD diffractometer	3756 independent reflections
Radiation source: fine-focus sealed tube	3052 reflections with *I* > 2σ(*I*)
graphite	*R*_int_ = 0.033
ω and φ scans	θ_max_ = 27.4°, θ_min_ = 2.2°
Absorption correction: multi-scan (*SORTAV*; Blessing, 1997)	*h* = −11→11
*T*_min_ = 0.971, *T*_max_ = 0.986	*k* = −23→18
6489 measured reflections	*l* = −13→13

### Refinement

**Table:**

Refinement on *F*^2^	Primary atom site location: structure-invariant direct methods
Least-squares matrix: full	Secondary atom site location: difference Fourier map
*R*[*F*^2^ > 2σ(*F*^2^)] = 0.049	Hydrogen site location: inferred from neighbouring sites
*wR*(*F*^2^) = 0.112	H-atom parameters constrained
*S* = 1.09	*w* = 1/[σ^2^(*F*_o_^2^) + (0.0212*P*)^2^ + 2.3993*P*] where *P* = (*F*_o_^2^ + 2*F*_c_^2^)/3
3756 reflections	(Δ/σ)_max_ < 0.001
244 parameters	Δρ_max_ = 0.30 e Å^−^^3^
0 restraints	Δρ_min_ = −0.42 e Å^−^^3^

### Special details

**Table:**

Geometry. All esds (except the esd in the dihedral angle between two l.s. planes) are estimated using the full covariance matrix. The cell esds are taken into account individually in the estimation of esds in distances, angles and torsion angles; correlations between esds in cell parameters are only used when they are defined by crystal symmetry. An approximate (isotropic) treatment of cell esds is used for estimating esds involving l.s. planes.
Refinement. Refinement of *F*^2^ against ALL reflections. The weighted *R*-factor wR and goodness of fit *S* are based on *F*^2^, conventional *R*-factors *R* are based on *F*, with *F* set to zero for negative *F*^2^. The threshold expression of *F*^2^ > σ(*F*^2^) is used only for calculating *R*-factors(gt) etc. and is not relevant to the choice of reflections for refinement. *R*-factors based on *F*^2^ are statistically about twice as large as those based on *F*, and *R*- factors based on ALL data will be even larger.

### Fractional atomic coordinates and isotropic or equivalent isotropic displacement parameters (Å^2^)

**Table:**

	*x*	*y*	*z*	*U*_iso_*/*U*_eq_	
S1	0.16733 (7)	0.13166 (4)	0.14038 (7)	0.03295 (17)	
S2	0.38728 (7)	0.12529 (3)	0.66150 (6)	0.02728 (15)	
O1	0.2772 (2)	0.12845 (12)	0.0444 (2)	0.0478 (5)	
O2	0.1836 (2)	0.18787 (11)	0.2374 (2)	0.0429 (5)	
O3	−0.0103 (2)	−0.04351 (11)	0.2382 (2)	0.0442 (5)	
O4	0.4221 (2)	0.07537 (10)	0.76644 (19)	0.0359 (4)	
O5	0.5094 (2)	0.14955 (11)	0.5886 (2)	0.0398 (5)	
O6	0.0076 (2)	0.11239 (11)	0.48394 (19)	0.0383 (5)	
O7	0.3567 (2)	0.03938 (11)	0.37697 (18)	0.0383 (5)	
N1	0.1573 (2)	0.04988 (12)	0.2123 (2)	0.0310 (5)	
N2	0.2511 (2)	0.09045 (12)	0.5604 (2)	0.0290 (5)	
C1	−0.2366 (3)	0.05097 (15)	0.0789 (3)	0.0346 (6)	
H1	−0.2855	0.0083	0.1071	0.042*	
C2	−0.3127 (3)	0.10214 (17)	0.0004 (3)	0.0411 (7)	
H2	−0.4159	0.0948	−0.0245	0.049*	
C3	−0.2408 (4)	0.16386 (17)	−0.0425 (3)	0.0438 (7)	
H3	−0.2948	0.1972	−0.0988	0.053*	
C4	−0.0923 (3)	0.17795 (15)	−0.0053 (3)	0.0383 (6)	
H4	−0.0437	0.2208	−0.0328	0.046*	
C5	−0.0177 (3)	0.12689 (14)	0.0738 (3)	0.0291 (5)	
C6	−0.0865 (3)	0.06398 (13)	0.1153 (3)	0.0278 (5)	
C7	0.0161 (3)	0.01604 (14)	0.1959 (3)	0.0302 (5)	
C8	0.2930 (3)	0.01150 (16)	0.2583 (3)	0.0381 (7)	
H8A	0.2690	−0.0406	0.2702	0.046*	
H8B	0.3673	0.0149	0.1904	0.046*	
C9	0.0236 (3)	0.24265 (15)	0.6728 (3)	0.0340 (6)	
H9	−0.0741	0.2401	0.6307	0.041*	
C10	0.0619 (3)	0.29787 (15)	0.7627 (3)	0.0381 (6)	
H10	−0.0114	0.3329	0.7828	0.046*	
C11	0.2047 (3)	0.30251 (15)	0.8233 (3)	0.0358 (6)	
H11	0.2281	0.3411	0.8831	0.043*	
C12	0.3143 (3)	0.25158 (14)	0.7980 (3)	0.0342 (6)	
H12	0.4124	0.2543	0.8393	0.041*	
C13	0.2738 (3)	0.19682 (14)	0.7098 (3)	0.0281 (5)	
C14	0.1321 (3)	0.19172 (14)	0.6466 (2)	0.0282 (5)	
C15	0.1155 (3)	0.12903 (14)	0.5542 (2)	0.0284 (5)	
C16	0.2762 (3)	0.02374 (14)	0.4899 (3)	0.0331 (6)	
H16A	0.3342	−0.0108	0.5477	0.040*	
H16B	0.1788	0.0009	0.4628	0.040*	

### Atomic displacement parameters (Å^2^)

**Table:**

	*U*^11^	*U*^22^	*U*^33^	*U*^12^	*U*^13^	*U*^23^
S1	0.0283 (3)	0.0322 (3)	0.0387 (4)	−0.0050 (3)	0.0046 (3)	−0.0050 (3)
S2	0.0211 (3)	0.0322 (3)	0.0282 (3)	0.0023 (2)	−0.0006 (2)	−0.0019 (3)
O1	0.0381 (11)	0.0540 (13)	0.0533 (14)	−0.0084 (10)	0.0177 (10)	−0.0029 (11)
O2	0.0383 (11)	0.0362 (10)	0.0539 (13)	−0.0069 (9)	0.0005 (10)	−0.0149 (10)
O3	0.0501 (12)	0.0346 (10)	0.0474 (13)	−0.0041 (9)	−0.0018 (10)	0.0132 (9)
O4	0.0347 (10)	0.0398 (10)	0.0324 (10)	0.0094 (8)	−0.0047 (8)	0.0011 (8)
O5	0.0256 (9)	0.0499 (12)	0.0447 (12)	−0.0026 (8)	0.0081 (8)	−0.0036 (10)
O6	0.0296 (9)	0.0494 (11)	0.0347 (11)	0.0017 (8)	−0.0086 (8)	0.0000 (9)
O7	0.0296 (10)	0.0552 (12)	0.0295 (10)	0.0028 (9)	−0.0031 (8)	−0.0124 (9)
N1	0.0264 (11)	0.0316 (11)	0.0342 (12)	0.0032 (9)	−0.0036 (9)	−0.0028 (10)
N2	0.0243 (10)	0.0346 (11)	0.0274 (11)	0.0034 (9)	−0.0029 (8)	−0.0040 (9)
C1	0.0313 (13)	0.0371 (14)	0.0348 (15)	−0.0031 (11)	−0.0024 (11)	−0.0061 (12)
C2	0.0336 (14)	0.0521 (17)	0.0358 (15)	0.0082 (13)	−0.0112 (12)	−0.0132 (13)
C3	0.0567 (19)	0.0400 (16)	0.0335 (16)	0.0168 (14)	−0.0064 (14)	−0.0013 (13)
C4	0.0509 (17)	0.0316 (13)	0.0327 (15)	0.0032 (12)	0.0043 (13)	0.0036 (12)
C5	0.0295 (13)	0.0292 (12)	0.0287 (13)	0.0000 (10)	0.0025 (10)	−0.0010 (10)
C6	0.0274 (12)	0.0270 (12)	0.0285 (13)	0.0005 (10)	−0.0008 (10)	−0.0029 (10)
C7	0.0335 (13)	0.0297 (12)	0.0270 (13)	0.0009 (11)	−0.0023 (11)	−0.0024 (11)
C8	0.0315 (14)	0.0471 (16)	0.0345 (15)	0.0121 (12)	−0.0083 (12)	−0.0124 (13)
C9	0.0281 (13)	0.0375 (14)	0.0366 (15)	0.0058 (11)	0.0047 (11)	0.0064 (12)
C10	0.0405 (15)	0.0341 (14)	0.0406 (16)	0.0108 (12)	0.0090 (13)	0.0031 (12)
C11	0.0431 (15)	0.0312 (13)	0.0339 (14)	0.0012 (11)	0.0081 (12)	−0.0028 (11)
C12	0.0340 (14)	0.0346 (13)	0.0341 (15)	−0.0026 (11)	0.0041 (12)	−0.0026 (12)
C13	0.0229 (11)	0.0323 (12)	0.0296 (13)	0.0021 (10)	0.0046 (10)	0.0020 (11)
C14	0.0272 (12)	0.0311 (12)	0.0266 (13)	0.0007 (10)	0.0036 (10)	0.0073 (10)
C15	0.0255 (12)	0.0341 (13)	0.0256 (12)	0.0022 (10)	0.0019 (10)	0.0060 (11)
C16	0.0328 (13)	0.0329 (13)	0.0332 (14)	−0.0004 (11)	−0.0017 (11)	−0.0032 (11)

### Geometric parameters (Å, °)

**Table:**

S1—O1	1.429 (2)	C3—C4	1.382 (4)
S1—O2	1.431 (2)	C3—H3	0.9500
S1—N1	1.676 (2)	C4—C5	1.381 (4)
S1—C5	1.750 (3)	C4—H4	0.9500
S2—O5	1.4280 (19)	C5—C6	1.387 (3)
S2—O4	1.4287 (19)	C6—C7	1.482 (3)
S2—N2	1.673 (2)	C8—H8A	0.9900
S2—C13	1.748 (2)	C8—H8B	0.9900
O3—C7	1.205 (3)	C9—C14	1.384 (3)
O6—C15	1.205 (3)	C9—C10	1.396 (4)
O7—C8	1.402 (3)	C9—H9	0.9500
O7—C16	1.424 (3)	C10—C11	1.385 (4)
N1—C7	1.407 (3)	C10—H10	0.9500
N1—C8	1.454 (3)	C11—C12	1.390 (4)
N2—C15	1.401 (3)	C11—H11	0.9500
N2—C16	1.445 (3)	C12—C13	1.382 (4)
C1—C2	1.386 (4)	C12—H12	0.9500
C1—C6	1.390 (3)	C13—C14	1.388 (3)
C1—H1	0.9500	C14—C15	1.489 (4)
C2—C3	1.387 (5)	C16—H16A	0.9900
C2—H2	0.9500	C16—H16B	0.9900
			
O1—S1—O2	117.27 (13)	O3—C7—N1	123.5 (2)
O1—S1—N1	108.66 (12)	O3—C7—C6	127.6 (2)
O2—S1—N1	110.47 (12)	N1—C7—C6	108.8 (2)
O1—S1—C5	113.86 (13)	O7—C8—N1	112.8 (2)
O2—S1—C5	110.97 (12)	O7—C8—H8A	109.0
N1—S1—C5	92.88 (11)	N1—C8—H8A	109.0
O5—S2—O4	116.83 (12)	O7—C8—H8B	109.0
O5—S2—N2	110.41 (12)	N1—C8—H8B	109.0
O4—S2—N2	109.43 (12)	H8A—C8—H8B	107.8
O5—S2—C13	112.74 (12)	C14—C9—C10	118.1 (3)
O4—S2—C13	112.00 (12)	C14—C9—H9	120.9
N2—S2—C13	92.81 (11)	C10—C9—H9	120.9
C8—O7—C16	115.2 (2)	C11—C10—C9	121.3 (3)
C7—N1—C8	123.2 (2)	C11—C10—H10	119.4
C7—N1—S1	114.55 (17)	C9—C10—H10	119.4
C8—N1—S1	120.62 (19)	C10—C11—C12	121.0 (3)
C15—N2—C16	124.5 (2)	C10—C11—H11	119.5
C15—N2—S2	115.34 (17)	C12—C11—H11	119.5
C16—N2—S2	120.12 (17)	C13—C12—C11	116.8 (3)
C2—C1—C6	117.9 (3)	C13—C12—H12	121.6
C2—C1—H1	121.0	C11—C12—H12	121.6
C6—C1—H1	121.0	C12—C13—C14	123.1 (2)
C1—C2—C3	121.1 (3)	C12—C13—S2	126.7 (2)
C1—C2—H2	119.5	C14—C13—S2	110.25 (19)
C3—C2—H2	119.5	C9—C14—C13	119.6 (2)
C4—C3—C2	121.5 (3)	C9—C14—C15	126.9 (2)
C4—C3—H3	119.3	C13—C14—C15	113.5 (2)
C2—C3—H3	119.3	O6—C15—N2	123.8 (2)
C5—C4—C3	117.0 (3)	O6—C15—C14	128.1 (2)
C5—C4—H4	121.5	N2—C15—C14	108.1 (2)
C3—C4—H4	121.5	O7—C16—N2	109.4 (2)
C4—C5—C6	122.5 (2)	O7—C16—H16A	109.8
C4—C5—S1	127.1 (2)	N2—C16—H16A	109.8
C6—C5—S1	110.41 (19)	O7—C16—H16B	109.8
C5—C6—C1	120.0 (2)	N2—C16—H16B	109.8
C5—C6—C7	113.2 (2)	H16A—C16—H16B	108.2
C1—C6—C7	126.8 (2)		
			
O1—S1—N1—C7	−118.8 (2)	C1—C6—C7—O3	3.8 (5)
O2—S1—N1—C7	111.19 (19)	C5—C6—C7—N1	2.2 (3)
C5—S1—N1—C7	−2.5 (2)	C1—C6—C7—N1	−178.7 (2)
O1—S1—N1—C8	47.0 (2)	C16—O7—C8—N1	71.9 (3)
O2—S1—N1—C8	−83.0 (2)	C7—N1—C8—O7	−118.3 (3)
C5—S1—N1—C8	163.4 (2)	S1—N1—C8—O7	77.1 (3)
O5—S2—N2—C15	−114.79 (19)	C14—C9—C10—C11	0.8 (4)
O4—S2—N2—C15	115.27 (19)	C9—C10—C11—C12	−1.0 (4)
C13—S2—N2—C15	0.8 (2)	C10—C11—C12—C13	0.2 (4)
O5—S2—N2—C16	67.1 (2)	C11—C12—C13—C14	0.8 (4)
O4—S2—N2—C16	−62.9 (2)	C11—C12—C13—S2	−179.1 (2)
C13—S2—N2—C16	−177.4 (2)	O5—S2—C13—C12	−66.3 (3)
C6—C1—C2—C3	1.1 (4)	O4—S2—C13—C12	67.9 (3)
C1—C2—C3—C4	−2.2 (4)	N2—S2—C13—C12	−179.8 (2)
C2—C3—C4—C5	1.6 (4)	O5—S2—C13—C14	113.83 (19)
C3—C4—C5—C6	−0.1 (4)	O4—S2—C13—C14	−111.97 (19)
C3—C4—C5—S1	−177.6 (2)	N2—S2—C13—C14	0.3 (2)
O1—S1—C5—C4	−66.8 (3)	C10—C9—C14—C13	0.1 (4)
O2—S1—C5—C4	68.1 (3)	C10—C9—C14—C15	−179.7 (2)
N1—S1—C5—C4	−178.6 (3)	C12—C13—C14—C9	−0.9 (4)
O1—S1—C5—C6	115.5 (2)	S2—C13—C14—C9	179.0 (2)
O2—S1—C5—C6	−109.6 (2)	C12—C13—C14—C15	178.9 (2)
N1—S1—C5—C6	3.7 (2)	S2—C13—C14—C15	−1.2 (3)
C4—C5—C6—C1	−0.9 (4)	C16—N2—C15—O6	−3.9 (4)
S1—C5—C6—C1	176.9 (2)	S2—N2—C15—O6	178.1 (2)
C4—C5—C6—C7	178.2 (2)	C16—N2—C15—C14	176.5 (2)
S1—C5—C6—C7	−3.9 (3)	S2—N2—C15—C14	−1.5 (3)
C2—C1—C6—C5	0.4 (4)	C9—C14—C15—O6	2.0 (4)
C2—C1—C6—C7	−178.6 (3)	C13—C14—C15—O6	−177.8 (3)
C8—N1—C7—O3	12.8 (4)	C9—C14—C15—N2	−178.5 (2)
S1—N1—C7—O3	178.2 (2)	C13—C14—C15—N2	1.7 (3)
C8—N1—C7—C6	−164.8 (2)	C8—O7—C16—N2	−128.4 (2)
S1—N1—C7—C6	0.6 (3)	C15—N2—C16—O7	101.2 (3)
C5—C6—C7—O3	−175.2 (3)	S2—N2—C16—O7	−80.8 (2)

### Hydrogen-bond geometry (Å, °)

**Table:**

*D*—H···*A*	*D*—H	H···*A*	*D*···*A*	*D*—H···*A*
C1—H1···O4^i^	0.95	2.39	3.307 (4)	162
C2—H2···O4^ii^	0.95	2.53	3.293 (3)	138
C8—H8B···O4^iii^	0.99	2.52	3.027 (3)	111
C8—H8A···O3	0.99	2.50	2.887 (4)	103

Symmetry codes: (i) −*x*, −*y*, −*z*+1; (ii) *x*−1, *y*, *z*−1; (iii) −*x*+1, −*y*, −*z*+1.
